# An Analysis of the Spatial Distribution of *Plasmodium sporozoites* and Effects of Climatic Correlates on Malaria Infection in Anyigba Town, Nigeria

**DOI:** 10.5539/gjhs.v6n1p115

**Published:** 2013-10-27

**Authors:** O. O. Ifatimehin, O. O. Falola, E. V. Odogbo

**Affiliations:** 1 Department of Geography and Planning, Kogi State University, Anyigba, Nigeria; 2 Department of Biological Sciences, Kogi State University, Anyigba, Nigeria

**Keywords:** incidence, prevalence, sanitation, ITN, risk, infectivity, sporozoites, climate

## Abstract

The infectivity of sporozoites on both mosquitoes and human is the major cause of malaria infection on its host, Man. Malaria infection had continued to blossom despite measures to curb it. Clinically diagnosed malaria data for 3 years, capture of mosquitoes for laboratory analysis to determining the infectivity of sporozoites, responses from the population on the number of episode of malaria in the last 60 days were all collected and generated, and also subjected to various analysis using methods accepted tools and methods. A fifteen weeks climatic data was also collected. It was discovered that malaria incidence of 467.2853/1000 persons is very high. This high rate is possible as out of every 10 mosquitoes in Anyigba, 4 are infected by sporozoites and can possibly transmit these sporozoites during blood feeding on the population. This is affirmed by the prevalence of malaria by 54.75% among Anyigba’s population. At p>001 (0.829), climatic variables and sporozoites rate showed a strong affinity with the prevalence of malaria. The risk map showed that the university community and the surrounding students’ lodges are areas of very high risk. Therefore, the populace is strongly advised to employed practicable measures such as regular environmental sanitation and the use of Insecticidal Treated Nets (ITN) in order to drastically address this epidemic.

## 1. Introduction

Malaria is one of the most devastating human diseases, with an estimates of about 300-500 million clinical cases every year with over 1.2 to 1.7 million deaths containing over one million children under 5years of age in African countries with over 90% of such cases in sub-saharan Africa (Pimenta et al., 1994; Lopez et al., 2006). Martens et al. (1999) singled out malaria as a vulnerable target which is a combination of vector (mosquitoes of the genus *Anopheles*) and parasites (*Plasmodium* spp) which affect thousands of people living in the world especially, tropical Africa.

The *Plasmodium* completes its complex developmental stages in adult mosquitoes, leading to the production of the infective form of the parasite, the sporozoite. The invasion of the salivary gland by sporozoite makes it more potent and infective in the transmission of the parasite to humans by mosquitoes (Touray et al., 1992). The complex interplay between the seasonal dynamics of the environment and condition of the environment also aids the interaction of parasite transmission and infectivity (Matuschewski et al., 2002; Ifatimehin et al., 2012). Arruda et al. (1998) affirmed that sporozoite rate is higher by 66.1% among rural population than in the urban population. Oyewole et al. (2005) posited that indoor sporozoite rate is higher in exophagous mosquitoes in the dry season than in the endophagous mosquitoes (indoor) while in the wet season sporozoite is more pronounced indoor than outdoor. The disease triad (Agent, Host and Environment) show the interactive nature of the variables of the physical, environmental, medical/socio-economic importance of the disease with the survival of the causative agent *Plasmodium* spp (sporozoites) and Vectors (mosquitoes) and how it gets to the host (Man) (Rockett, 1999).

Malaria incidence and prevalence is reported to be on the increase despite all measures of intervention put forward. There are over 100 million people at risk every year in Nigeria and indeed, it is estimated that about 50% of adult population in Nigeria experiences at least one episode yearly while children under the age five have up to 2-4 attacks of malaria annually (WHO, 2005). Therefore, this study was carried out in order to provide baseline information on the increasing infection of female *Anopheles* mosquitoes with *Plasmodium* sporozoite and how it relates to the increasing prevalence and incidence of malaria among the teeming population of Anyigba town.

## 2. Materials and Methods

### 2.1 Study Area

**Figure 1:**
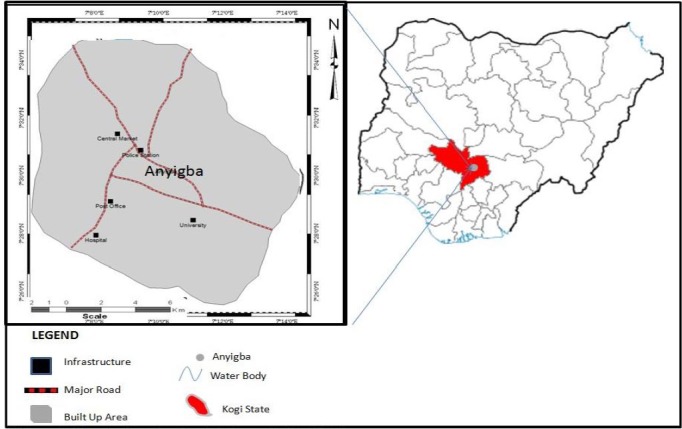
Nigeria showing Anyigba town in Kogi State

Anyigba is a town in Dekina Local Government Area in Kogi State located between latitudes 7°15'N-7°29'N and longitudes 7°11'E – 7°32'E (Figure 1). With an average altitude of 385 meters above sea level and total land mass area of 420 Sq.km^2^ has an estimated population of 189,976 persons (Ifatimehin, 2012). The study area falls within the tropical wet and dry (Aw) climatic region and the derived savanna. The annual mean rainfall and temperature are 1250mm and 25°C. The land use and economy of the area is predominantly agrarian in the first instance but fast changing because of the transformation initiated in the economic landscape by the presence of the State University (Ifatimehin & Ufuah, 2006). The level of sanitation in the present Anyigba is very poor as no artificial drainage system exist, indiscriminate refuse and sewage disposal and bushy environments: all these serve as breeding sites for mosquitoes (Ifatimehin & Musa, 2009; Ifatimehin et al., 2009).

### 2.2 Collection of Clinical Data

A 3 year (2010-2012) clinical data was collected from identified health facilities in the study area. Diagnosed cases and information about the surrounding the location (Neighborhoods) of each patient were also collected. This is to sort the number of diagnosed and treated patients within the three differentiated areas of study

### 2.3 Collection of Climatic Data

A fifteen weeks climatic data comprising of rainfall amount, minimum and maximum temperatures and humidity were collected from the university weather stations. These climatic data were collected during the period of the study.

### 2.4 Sampling and Examination of Mosquitoes

Mosquitoes were collected at the beginning of the new moon using scoop net on the flying ones; collection was carried out in areas with predominantly dense population (Anyigba community, University environment and students village – resident areas). The Global Positioning System (GPS) Receiver was used to generate a 2-dimensional (Latitude and Longitude) reading for each point of mosquitoes capture in the identified 25 points. The mosquitoes were placed in a container with wet cotton wool and carried to the laboratory. The mosquitoes were made unconscious by gently shaking the container. Identification and confirmation of species were made using the key of lavereni. Landing catch comparison was made between the collections from outside and inside the houses.

The wings and legs of the mosquitoes identified were removed. Each of the mosquitoes were placed on a slide with a small drop of saline solution around the head which was clearly cut off near the thorax. The dissection was done under a x10 lens objective of the microscope. Illumination by transmitted light was adjusted so that the glands showed up clearly against the background. A Shute’s needle was held in the left hand and placed flat across the thorax. Very gentle pressure was exerted which caused the two trilobed salivary glands to pop out from the end of the neck, accompanied by fat globules, some muscle fibres and other tissues. When the glands have been located, under x10 objective lens of the microscope, they were freed from the debris and pulled to the edge of the slide. A drop of saline solution was added and a cover slip was gently placed on it. The glands were by pressing down the cover slip with a dissecting needle. The ruptured glands were then examined under a x40 objective lens of the microscope for the sickle-shaped motile sporozoites.

### 2.5 Administration of Questionnaire

A sample population of 450 respondents within the three differentiated areas (Anyigba community, the University and the Students’ lodges for the study was identified and was given copies of the questionnaire to ascertain the occurrence of malaria infection within the last 60 days before the study.

**Table 1:** Number of infected mosquitoes with sporozoites and questionnaire administered

Sample site	S/N	Exophagic Mosquitoes (Outdoor) feeders	Endophagic Mosquitoes (Indoor) feeders	No. of Mosquitoes		Copies of Questionnaire	
				Dissected	+ve pools	Distributed	Returned
Anyigba Community	1	Ofejikpi		35	16	20	15
	2	OLS Area		50	23	25	22
	3	Atenegoma		45	26	25	21
	4	Market Area		40	20	25	24
	5		Unity Square	42	18	15	13
	6		Aabuja	23	11	15	12
	7		Iji	45	19	25	23
University Environment	8	Idah Avenue		30	12	25	21
	9	B-Block		20	8	10	10
	10	Professorial Quarters		34	17	15	15
	11	Dekina Avenue		45	27	20	19
	12	NTA Area		30	12	10	10
	13		Ocheja Hostel	30	22	20	20
	14		Male Hostel	15	3	20	20
	15		Female Hostel	22	4	20	20
	16		Dangana hostel	34	18	20	20
	17		Inikpi Hostel	29	11	20	20
Students Village	18	Oxford lodge		28	12	20	20
	19	Millionaire Quarters		32	11	15	15
	20	Passover Lodge		35	21	20	15
	21	Eleojo Lodge		32	13	20	17
	22		Yaso Lodge	32	7	10	8
	23		Victory Lodge	30	8	10	8
	24		London base	28	9	10	9
	25		Trinity Lodge	25	11	15	14
**TOTAL**				**811**	**359**	**450**	**411**

### 2.6 Analysis

The incidence rate was estimated using the 3 years clinical diagnosed data retrieved from the various health centres in the study area. The sporozoites rates were as well determined. Pearson correlation and linear regression analysis were used to establish the level of relationship between the climatic variables, sporozoites and prevalence rates. Kriging a spatial statistical tool was also employed to determine the risk map of the study area. The variables identified where buffered using the flight distance of 1.5km of adult mosquitoes from breeding sites to blood feeding points. ArcGIS 9.2 and SPSS 17 softwares were used for these operations.

## 3. Results

### 3.1 Malaria Incidence Rate

Malaria infection among the population of Anyigba is alarming as shown in Table 2 where the incidence rate is above 460. This implies that out of every 1000 persons, 467 had suffered the ailment. This alarming index may be as a result of certain factors within the town that may encourage the infection among the population. The table also shows that the infection is on the increase as it increases successively in 2011 and 2013. Within the three years in reference, annual increase rate of 403/yr also implies that addition 403 cases are reported every other year.

**Table 2:** Malaria Incidence rate of Anyigba

Total Population	Annual clinical diagnosed malaria cases					
	2010	2011	2012	Total	Annual increase rate/yr	Incidence Rate/1000 persons
189,976	29066	29432	30275	88773	403	467.2853

Authors’ Fieldwork, 2012

The high incidence rate in the town may be as result of the environment been conducive for the breeding of mosquitoes and also the presence of the parasite (*Plasmodium* spp) within the environment. This parasite is transmitted to humans through the vector; thefemale *Anopheles* mosquito. The population may also be contributing to the suitability of the environment to support the growth and infection of the parasite on the vector.

### 3.2 Sporozoites Rate in Anyigba

The parasite life cycle in the mosquitoes provides the transformation of *Plasmodium* into the infective form of the parasite: the sporozoites. The sporozoites rate shown in table 3 indicates that 73.33% of the mosquitoes found around Ocheja Hostel in the University are infected with sporozoites while Female hostel (18.18%) has the least infected mosquitoes. Out of the 811 mosquitoes captured, 44.27% are infected with the parasite, and this is distributed across the town withAnyigba community having the highest infected mosquitoes (47.50%) and the Students’ Lodges having the least (38.02%). The University Community had about 46.37% infected mosquitoes.

**Table 3:** Sporozoites rates of Anyigba town

S/N	Sites of Capture		No. of Mosquitoes		Sporozoites Rate (%)
			Dissected	+ve pools
1	Anyigba Community	Ofejikpi	35	16	45.71
2		OLS Area	50	23	46.00
3		Atenegoma	45	26	57.78
4		Market Area	40	20	50.00
5		Unity Square	42	18	42.86
6		Aabuja	23	11	47.82
7		Iji	45	19	42.22
***Sub total***			***280***	***133***	***47.5***
8	University Community	Idah Avenue	30	12	40.00
9		B-Block	20	8	40.00
10		Professorial Quarters	34	17	50.00
11		Dekina Avenue	45	27	60.00
12		NTA Area	30	12	40.00
13		Ocheja Hostel	30	22	73.33
14		Male Hostel	15	3	20.00
15		Female Hostel	22	4	18.18
16		Dangana hostel	34	18	52.94
17		Inikpi Hostel	29	11	37.93
***Sub-total***			***289***	***134***	***46.37***
18	Students Lodges	Oxford lodge	28	12	42.86
19		Millionaire Quarters	32	11	34.38
20		Passover Lodge	35	21	60.00
21		Eleojo Lodge	32	13	40.63
22		Yaso Lodge	32	7	21.88
23		Victory Lodge	30	8	26.67
24		London base	28	9	32.14
25		Trinity Lodge	25	11	44.00
***Sub-total***			***242***	***92***	***38.02***
**Total**			**811**	**359**	**44.27**

Source: Authors’ Fieldwork, 2012

Of the 811 mosquitoes captured, 456 were captured outside the rooms, while 355 were captured indoors: in living rooms, toilets and bathrooms. 46.72% of the exophagic mosquitoes are infected with the parasites while 38.33% of the endophagic mosquitoes are infected. Dekina avenue and Passover Lodge had the highest (60%) sporozoites rate among the exophagic mosquitoes examined. Atenegoma had 57.77%, Market area and Professorial quarters 50.00% each, while other sites of mosquitoes collection had below 50.00% with Millionaire quarters having the least (34.38%). For the endophagic mosquitoes studied, Ocheja Hostel has the highest rate of infected mosquitoes (73.33%) in its rooms, toilets and bathrooms. Dangana Hostel had 52.94% while other sites had below the 50% mark with the female hostel having the least (18.18%). Exophagic mosquitoes are more potent and infective for the transmission of the sporozoites to their victims.

**Table 4:** Exophagic and Endophagic distribution of Mosquitoes

**s/n**	**Exophagic Mosquitoes**			**Endophagic Mosquitoes**		
	**Sites of collection**		**Sporozoite rate (%)**	**Sites of collection**		**Sporozoite rate (%)**
1	Anyigba Community	Ofijikpi	45.71	Anyigba Community	Unity Square	42.86
2		OLS Area	46.00		AaBuja	47.83
3		Atenegoma	57.77		Iji	42.22
4		Market Area	50.00			
5	University Environment	Staff Quarters (Obasanjo Extension)	40.00	University Environment	Ocheja Hostel	73.33
6		B-Block	40.00		Male Hostel	20.00
7		Professorial Quarters	50.00		Female Hostel	18.18
8		Dekina Avenue	60.00		Dangana Hostel	52.94
9		NTA Area	40.00		Inikpi Hostel	37.93
10	Students Lodges	Oxford Lodge	42.86	Students Lodges	Yaso Lodge	21.86
					Victory Lodge	26.67
11		Millionnaire Quarters	34.38		London Base	32.14
12		Passover Lodge	60.00		Trinity Lodge	44.00
13		Eleojo Lodge	40.63		
**Average Sporozoite Rate (%)**			**46.72**	**Average Sporozoite Rate (%)**		**38.33**

Source: Authors’ Fieldwork, 2012

### 3.3 Prevalence Rate of Malaria in Anyigba

About 54.75% of the respondents claimed they had at least one episode of malaria infection in the last 60 days. This also coincided with the prevalence rate. The university community had the highest prevalence rate of 57.71%, with the NTA area (80.00%), Dangana (65.00%), Professorial quarters, Inikpi hostel, Female Hostel and Ocheja Hostel all having60.00% prevalence and the Staff quarters (52.38%) all having above 50% prevalence rate. Anyigba community recorded 48.46% and the students’ lodges 57.55%. This implies that the university community and the students’ lodges had above 50% prevalence rate.

**Table 5:** Prevalence rate of Malaria in Anyigba

S/N	Sites of Capture		No. of respondents		Prevalence Rate of Malaria (%)
			Total No.	No. infected with Malaria
1	Anyigba Community	Ofejikpi	15	7	46.67
2		OLS Area	22	9	40.91
3		Atenegoma	21	12	57.14
4		Market Area	24	10	41.67
5		Unity Square	13	7	53.85
6		Abuja	12	5	41.67
7		Iji	23	13	56.52
***Sub total***			***130***	***63***	***48.46***
8	University Community	Staff quarters	21	11	52.38
9		B-Block	10	4	40.00
10		Professorial Quaters	15	9	60.00
11		Dekina Quarters	19	11	57.89
12		NTA Area	10	8	80.00
13		Ocheja Hostel	20	12	60.00
14		Male Hostel	20	9	45.00
15		Female Hostel	20	12	60.00
16		Dangana hostel	20	13	65.00
17		Inikpi Hostel	20	12	60.00
***Sub-total***			***175***	***101***	***57.71***
18	Students Lodges	Oxford lodge	20	11	55.00
19		Millionaire Quarters	15	8	53.33
20		Passover Lodge	15	11	73.33
21		Eleojo Lodge	17	11	64.71
22		Yaso Lodge	8	4	50.00
23		Victory Lodge	8	3	37.50
24		London base	9	5	55.56
25		Trinity	14	8	57.14
***Sub-total***			***106***	***61***	***57.55***
**Total**			**411**	**225**	**54.75**

### 3.4 Relationship between Malaria Prevalence, Sporozoites Rate and Climatic Variables in Anyigba

**Figure 2:**
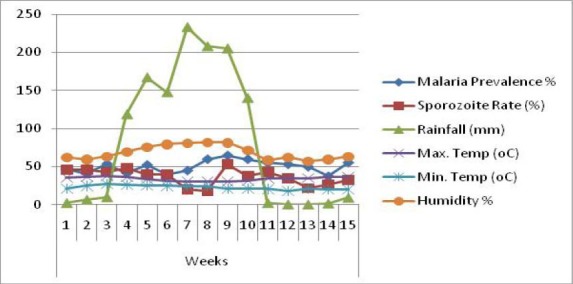
15 weeks Climatic pattern, sporozoites rates and Malaria Prevalence rate in Anyigba

Certainly, it is expected that blood feeding of female *Anopheles* mosquitoes take place more often at night and indoors. Any human bitten by infected mosquito is expected to be infected with the parasite which invariable may lead to malaria infection. Changes in temperature and humidity could impact on the breeding and feeding, maturation and the survival of mosquitoes, which consequently lead to changes in their distribution and habitation. Figure 2 shows the relationship between climatic variables (rainfall amount, maximum and minimum temperatures, and humidity), sporozoites rate and malaria prevalence.

From Table 6, all the variables showed a strong positive relationship among the variables. At p>0.01, the Pearson correlation coefficients ranges from a minimum of 0.616 between malaria prevalence rate and sporozoites rates to the maximum of 0.819 between malaria prevalence rate and maximum temperature. The sporozoites rate also shows a good and strong affinity with the climatic variables: maximum temperature (0.820), minimum temperature (0.805), rainfall (0.852) and humidity (0.792).

**Table 6:** Correlation Matrix between results

	Prevalence Rates	Sporozoites Rates	Rainfall	Max Temp	Min Temp	Humidity
**Prevalence Rates**	1.000					
**Sporozoites Rates**	.616	1.000				
**Rainfall**	.667	.852	1.000			
**Max Temp**	.819	.820	.868	1.000		
**Min Temp**	.724	.805	.916	.887	1.000	
**Humidity**	.781	.792	.768	.845	.776	1.000

Correlation is significant at the 0.01 level (2-tailed).

However, the coefficient of determination (r^2^) value of 0.687 implies that about 68.7% of the variation in malaria prevalence is caused by the individual climatic variables and the sporozoites rate. Therefore, the associations of all these variables as displayed in table 7 are accounted for in the linear regression equation.

**Table 7:** Coefficient of determination table

R	R^2^	Adjusted R^2^	Std Error of estimate	Change Statistics						
				R^2^ Change	F Change	df1	df2	Sig. F Change	Model	Standardized Coefficients (B/Beta)
.829	.687	.671	.35949	.687	42.654	5	97	.000	Constant	.750
									Sporozoites Rates (x_1_)	-.145
									Rainfall (x_2_)	-.212
									Max Temp (x_3_)	.916
									Min Temp (x_4_)	.175
									Humidity (x_5_)	.057


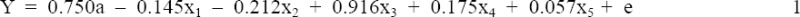


With this model therefore, the value of each of the variables (x_1_…… to x_5_) for a particular period can be used to project the severity of malaria at any point in time in further research works.

The attempt made by the populace to improve and fight the infection in the acquisition of Insecticidal Treated Nets as displayed in Table 8, shows that 75.6% of them owned the nets, but only 58.8% of those who owned the nets actually used. This implies that 41.2% of the do not use the nets. Above 50% of the populace in the university community do not use their ITN, and this also can possible accounted for the high prevalence of 57.71% of malaria in the community.

**Table 8:** Access to Insecticidal Treated Nets (ITN) among the Anyigba population

Sites	Owned			Do not owned	Sub total
	Usage	Do not use	Total		
**Anyigba Community**	24 (60%)	16 (40%)	40 (63.5%)	23 (36.5%)	63
**University Community**	37 (46.8%)	42 (53.2%)	79 (78.2%)	22 (21.8%)	101
**Students’ Lodges**	39 (76.5%)	12 (23.5%)	51 (83.6%)	10 (16.4%)	61
**Total**	**100 (58.8%)**	**70 (70%)**	**170 (75.6%)**	**55 (24.4%)**	**225**

Source: Authors’ Fieldwork, 2012

**Figure 3:**
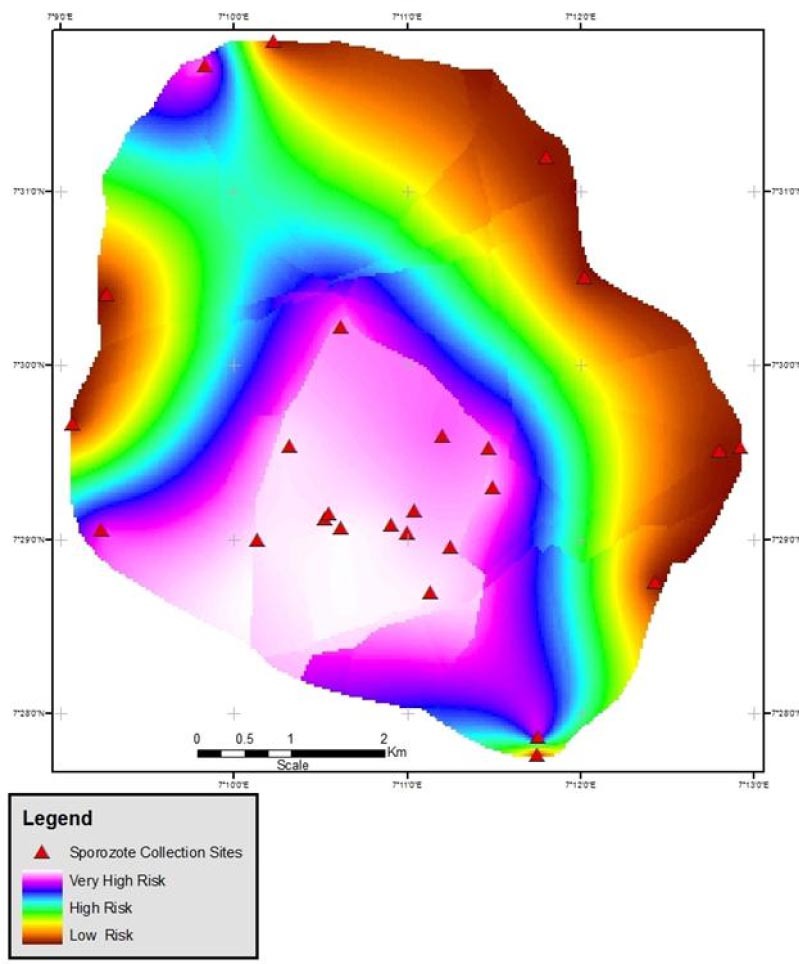
Plasmodium Sporozoites – Malaria Prevalence Risk Map of Anyigba

Therefore, the strong positive correlation value of 0.616 at p>0.01 value between sporozoites rate and prevalence is depicted in Figure 3 as areas of risk are shown.

## 4. Discussion

The three years diagnosed cases, shows that malaria incidence rate in the population of the town remained very high with epidemics in 2012, reaching 467. 2 per 1000 person as against the global average of 23.6 per 1000 person as reported by Castro et al. (2004). Therefore, Anyigba town is an endemic town as in every 1000 persons, 467 have had at least one episode of malaria infection within the year. As 403 new cases are reported annually, the transmission and distribution of the vector (Female *Anopheles* mosquito) is greatly influenced by climatic factors as reported by Minakawa et al. (2002) as well as the complex interplay of these factors on the sporozoites rates as posited by Kyes et al. (2001) in their works.

The 44.27% sporozoites rate in the study area although very small, implies that out of every 10 mosquitoes within Anyigba environment either indoor or outdoor, 4 are infected with sporozoites. This closely agrees with the work of Arruda et al. (1998) where sporozoites rate of 48.8% was recorded in the urban indigenous India population in the Amazon region of Brazil.

Source: Authors's Analysis, 2012

The total correlation coefficient of 0.829 at P>0.01 of all the variables used, compliments the high incidence rate (467 per 1000 persons), sporozoites rate (44.27%) and Prevalence rate (54.75%) of Malaria in the study area which attest to results from other studies in similar tropical regions as found out by Ifatimehin *et al*. (2012), Musa et al. (2012), Edillo et al (2008) and Shililu et al (2003). All the variables accounted for 68.7%, however, the remaining 31.3% may account for by sanitation, land use change and socio-economic factors as reported by Ifatimehin and Ogbe (2008), Oyewole et al. (2005), Leonardo et al. (2005), Shillu et al. (2003) and Minakawa et al. (2002).

The high malaria prevalence in the area can actually be attributed to the low use of ITN as only 75.6% of the population owned such nets. But only 58.8% of the population does make use of it, however only 44.4% of those who really owned the nets make use of them. This agrees with Ajadi *et al* (2011) as they reported that the use of ITN and other protective measures such as provision of screens to doors and windows, wearing of long sleeved shirts, trousers and gowns and others would reduce the exposure of the population to frequent mosquitoes bites.

Therefore, the high prevalence of malaria and its risk in Anyigba town is collectively dependent on the number of individuals suffering from the disease, climatic variables such as rainfall, maximum and minimum temperatures and humidity, low usage of ITN and environmental factors as supported by Ye-Ebiyo et al. (2000), Munga et al. (2006) and Krefis et al. (2010).

## 5. Conclusion

This study has revealed that the high malaria incidence rate in Anyigba is as a result of many factors especially climatic factors and the poor usage of ITN. This is most responsible for the high sporozoites rate among female *Anopheles* mosquitoes found within its environment both indoor and outdoor feeders and also the susceptibility of the population to malaria infection. The Anyigba community recorded the highest rate of prevalence indicating that everybody within the geographical metropolis are at risk of being infected despite the sporozoites rate recorded. Its high prevalence rate is observed to be as a result of poor town planning, compactness and crowdedness of the buildings, lack of drainages and as well as the sanitary condition of its environment.

Anyigba town is an endemic zone for malaria and therefore the following recommendations are proposed:

- Effort should be put in place to create a good awareness on the importance and effective and efficient use of Insecticidal Treated Nets;
- Regular and frequent sanitation exercises such as clearing of bushy environments, and proper waste disposal should be encourage;
- The use of window and door screens should be encouraged and
- Wearing of protective clothings when outside the house
